# Contribution of HOGG1 Ser^326^Cys Polymorphism to the Development of Prostate Cancer in Smokers: Meta-Analysis of 2779 Cases and 3484 Controls

**DOI:** 10.1371/journal.pone.0030309

**Published:** 2012-01-18

**Authors:** Bin Xu, Na Tong, Shu-Qiu Chen, Yu Yang, Xiao-Wen Zhang, Jin Liu, Xiang-Nong Hu, Guo-Zhu Sha, Ming Chen

**Affiliations:** 1 Department of Urology, Affiliated Zhongda Hospital, Southeast University, Nanjing, China; 2 Surgical Research Center, Medical School, Southeast University, Nanjing, China; 3 Department of Occupational Medicine and Environmental Health, Nanjing Medical University, Nanjing, China; Ohio State University Medical Center, United States of America

## Abstract

The HOGG1 gene catalyzes the excision of modified bases and removal of DNA damage adducts. It may play an important role in the prevention of carcinogenesis. Ser^326^Cys polymorphism localizes in exon 7 of the hOGG1 gene. It takes the form of an amino acid substitution, from serine to cysteine, in codon 326. Several epidemiological association studies have been conducted on this polymorphism and its relationship with the risk of prostate cancer. However, results have been conflicting. To resolve this conflict, we conducted a meta-analysis on the association between this polymorphism and prostate cancer, taking into account race, country, sources of controls, and smoking status. A total of nine studies covering 2779 cases and 3484 controls were included in the current meta-analysis. Although no significant association was found between hOGG1 Ser^326^Cys polymorphism and prostate cancer susceptibility in the pooled analysis, individuals with Ser/Cys+Cys/Cys genotypes were found to have greater risk of prostate cancer if they were also smokers (OR = 2.66, 95% CI = 1.58−4.47) rather than non-smokers (OR = 2.18, 95% CI = 1.13−4.19), compared with those with Ser/Ser genotype. In conclusion, our meta-analysis demonstrates that hOGG1 Ser^326^Cys polymorphism is a risk factor for prostate cancer in smokers. Further studies are needed to confirm this relationship.

## Introduction

Oxidative DNA damage is involved in carcinogenesis. It causes mutations that can inactivate tumor suppressor genes and activate oncogenes. The major form of DNA adduction induced by oxidative damage is 8-OH-dG (8-hydroxy-2-deoxyguanine) and increased 8-OH-dG formation in DNA results in mutagenesis and carcinogenesis [1]. A DNA glycosylase/apurinic-apyrimidinic lyase encoded by the human oxoguanine glycosylase 1 (hOGG1) gene catalyzes the excision of modified bases and removal of 8-OH-dG adducts and has been hypothesized to play an important role in the prevention of carcinogenesis [2].

Prostate cancer is most commonly-diagnosed malignancy in elderly men in developed countries, and the incidence increases every year. Deficient DNA repair mechanisms may play a role in the age-related increase in prostate cancer risk by allowing carcinogenic DNA damage events to accumulate uncorrected. Higher levels of 8-OH-dG and downregulation of hOGG1 also have been observed in benign prostatic hyperplasia (BPH) and prostate cancer.

Variations in the hOGG1 gene have been identified and the repair activities of the variant proteins have been evaluated in several studies. One of the most frequently analyzed variations is in exon 7 of the hOGG1 gene, which takes the form of a single amino acid substitution, from serine to cysteine at codon 326 (Ser^326^Cys, rs1052133). The hOGG1 protein encoded by the Ser^326^ allele showed much more DNA repair activity than that encoded by Cys^326^ allele *in vitro* [3]. There have also been several epidemiological studies concerning the association between this variation and the risk of prostate cancer. However, the results have not been conclusive. For example, Chen et al. found that carriers with the Cys^326^ allele had a significantly increased risk of prostate cancer, but Xu et al. found that subjects with Cys^326^ allele had a reduced risk of prostate cancer. For this reason, we conducted a meta-analysis on hOGG1 Ser^326^Cys polymorphism and the risk of prostate cancer, taking into account certain characteristics of the subjects and studies, such as race, country, source of controls and smoking status. We believe that this will help us better understand the risk of prostate cancer.

## Materials and Methods

### Publication search

The two online bibliographic databases (PubMed and Embase) were consulted with the following search strategy: “human oxoguanine glycosylase 1, hOGG1 or OGG1, hOGG or OGG,” “polymorphism or variant,” and “prostate cancer or prostate neoplasm” (last search was updated on Oct. 27, 2011). Original studies in English on hOGG1 polymorphism in prostate cancer were included; reviews, editorials, and letters were excluded. All the references of relevant reviews and eligible articles that our search retrieved were checked carefully.

### Inclusion criteria and data abstraction

Two investigators searched the literature and extracted data independently. The inclusion criteria were as follows: (i) Each study had to discuss or concern hOGG1 Ser^326^Cys polymorphism and the risk of prostate cancer. (ii) Each study had to use a case-control design. (iii) Each study had to contain information about available genotype frequency that could help technicians infer the results from the papers. For each of the eligible case-control studies, the following data were collected: the first author's last name, year of publication, country of origin of the subjects, race of the subjects, sources of controls, smoking status of the subjects, number of genotyped cases and controls, and Hardy-Weinberg equilibrium status.

### Statistical analysis

The association between hOGG1 different genotypes (including the heterozygote comparison (Ser/Cys vs. Ser/Ser) and the homozygote comparison (Cys/Cys vs. Ser/Ser), the dominant genetic model (Ser/Cys+Cys/Cys vs. Ser/Ser) and the recessive genetic model (Cys/Cys vs. Ser/Cys+Ser/Ser)) and susceptibility to prostate cancer was measured using the crude odds ratio (OR) with related 95% confidence interval (CI). A χ^2^-based Q-test was used to check the heterogeneity of the current study and determine the methods for calculating OR. If *P*>0.05 for a given *Q*-test indicated a lack of heterogeneity among the studies, then the summary ORs were calculated using the fixed-effects model (the Mantel-Haenszel method). Otherwise, the random effects model (DerSimonian and Laird method) was used. The significance of the pooled OR was determined using the *Z*-test, and *P*<0.05 was considered statistically significant. Subgroup analyses were also conducted on the basis of subject race, country, source of controls, and smoking status. The statistical power was calculated using PS software (http://biostat.mc.vanderbilt.edu/twiki/bin/view/Main/PowerSampleSize).

The publication bias was determined using Egger's linear regression test by visual inspection of the Funnel plot. All statistical analyses were performed using Stata 10.0 (StataCorp LP, College Station, TX, U.S.).

## Results

### Study characteristics

**Figure 1:**
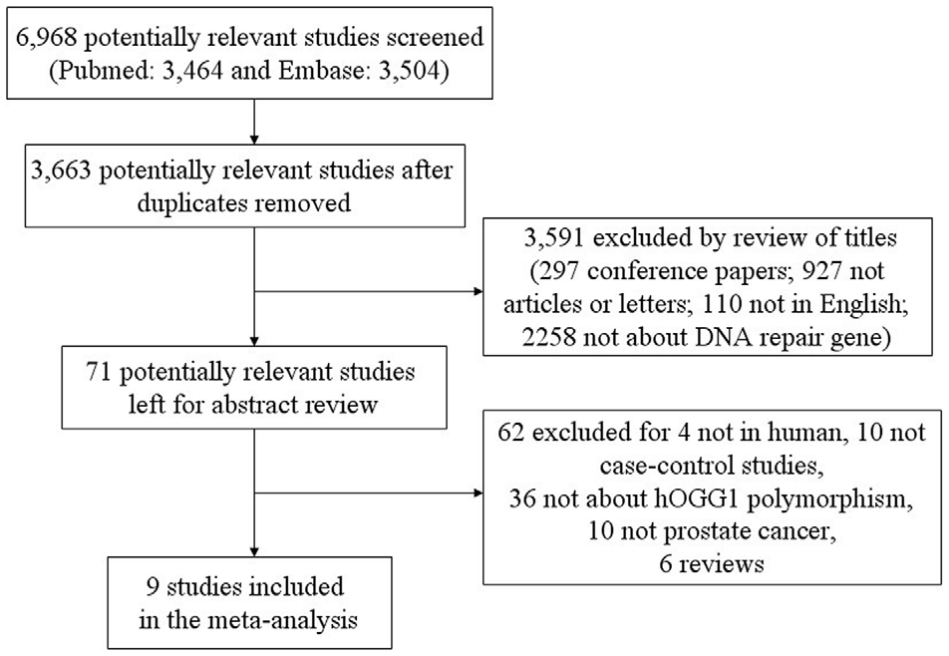
Included and excluded studies.

Characteristics of eligible studies are presented in Table 1 [4]–[12]. In all, 6,968 papers were retrieved by searching for the terms listed above (PubMed: 3,464 and Embase: 3,504). The study selection process is shown in Fig. 1. Nine studies with 2779 cases and 3484 controls were included in present meta-analysis [Bibr pone.0030309-Chen1] [Bibr pone.0030309-Xu1] [Bibr pone.0030309-Dhillon1] [Bibr pone.0030309-Yun1] [Bibr pone.0030309-Nock1] [Bibr pone.0030309-Nam1] [Bibr pone.0030309-Zhang1] [Bibr pone.0030309-Lavender1] [4–12].

**Table 1:** Main characteristics of all studies included in the meta-analysis.

First author	Year	Country	Race	Sources of controls	Smoking status considered	Case	Control	Cases			Controls			OR (95% CI)(Ser/Cys+Cys/Cys vs. Ser/Ser)	HWE
								Ser/Ser	Ser/Cys	Cys/Cys	Ser/Ser	Ser/Cys	Cys/Cys		
Chen [4]	2003	US	Caucasian	P	Y	84	251	49	29	6	185	63	3	2.00 (1.19–3.36)	Y
Dhillon [6]	2009	Australia	Caucasian	H	Y	116	131	38	57	21	69	50	12	2.28 (1.36–3.83)	Y
Lavender [11]	2010	US	African	P	N	194	646	132	58	4	452	173	21	1.09 (0.77–1.55)	Y
Mittal [12]	2011	North-India	Caucasian	P	N	195	250	98	83	14	136	100	14	1.18 (0.81–1.72)	Y
Nam [9]	2005	Canada	Mixed	H	N	996	1092	593	350	53	617	386	89	0.78 (0.52–1.18)	N
Nock [8]	2006	US	Mixed	P	N	439	478	280	135	24	305	142	31	0.88 (0.74–1.05)	N
Xu [5]	2002	US	Caucasian	P	N	298	174	182	106	10	96	63	15	0.78 (0.54–1.15)	Y
Yun [7]	2011	Korea	Asian	H	N	266	266	54	119	93	68	131	67	1.35 (0.90–2.02)	Y
Zhang [10]	2009	US	Mixed	P	N	191	196	126	61	4	118	71	7	1.13 (0.87–1.47)	Y

H, Hospital-based; P, Population-based.

Four studies had been conducted on Caucasians, one on Asians, one study on Africans, and three in mixed populations (Caucasians and African Americans). Six studies were population-based [Bibr pone.0030309-Chen1] [Bibr pone.0030309-Xu1] [Bibr pone.0030309-Nock1] [Bibr pone.0030309-Zhang1] [Bibr pone.0030309-Lavender1] [4,5,8,10–12]. The others were hospital-based [Bibr pone.0030309-Dhillon1] [Bibr pone.0030309-Yun1] [6,7,9]. In the hospital-based studies, controls were all selected from among patients without any sign of any type of cancer. The distribution of genotypes among controls was consistent with Hardy-Weinberg equilibrium in all studies except two [Bibr pone.0030309-Nock1] [8,9]. Two studies collected information on possible confounding factors like smoking status [Bibr pone.0030309-Chen1] [4,6]. In this way, the association between the Ser326Cys polymorphism and prostate cancer was separately evaluated among smokers and non-smokers. However, these two studies only provided the frequency data regarding Ser/Cys+Cys/Cys and Ser/Ser genotypes. For this reason, only a comparison of Ser/Cys+Cys/Cys vs. Ser/Ser was conducted.

### Meta-analysis

**Figure 2:**
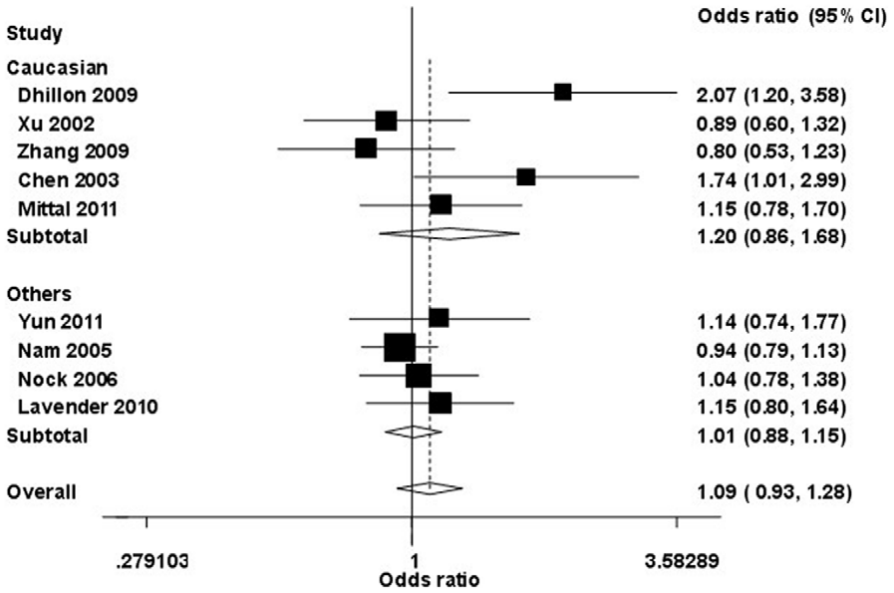
Forest plot of prostate cancer risk associated with hOGG1 polymorphism stratified by race. The squares and horizontal lines correspond to the study-specific OR and 95% CI. The area of the squares reflects the weight (inverse of the variance). The diamond represents the summary OR and 95% CI.

The main results of the current study on the association between hOGG1 Ser^326^Cys polymorphism and the risk of prostate cancer are shown in Table 2. Overall, no significant association with prostate cancer was observed using a random effects model in the comparisons of Ser/Cys to Ser/Ser (OR = 1.09, 95% CI: 0.93–1.28; *P*_heterogeneity_/*P* = 0.047/0.409), Cys/Cys vs. Ser/Ser (OR = 0.91, 95% CI: 0.55–1.52; *P*_heterogeneity_/*P* = <0.001/0.449), Ser/Cys+Cys/Cys vs. Ser/Ser (OR = 1.12, 95% CI: 0.92–1.37; *P*_heterogeneity_/*P* = 0.002/0.271), or Cys/Cys vs. Ser/Cys+Ser/Ser (OR = 1.03, 95% CI: 0.66–1.60; *P*_heterogeneity_/*P* = <0.001/0.913) in the pooled analysis (Fig. 2). We did not find any significant results when we stratified the studies by race, country, or source of the controls.

**Table 2:** Stratified analyses of the hOGG1 Ser326Cys polymorphism on prostate cancer risk.

Variables	n^a^	MAF	Power	Cases/controls	Ser/Cys vs. Ser/Ser		Cys/Cys vs. Ser/Ser		Ser/Cys+Cys/Cys vs. Ser/Ser		Cys/Cys vs. Ser/Cys+Ser/Ser	
					OR (95% CI)	*P* b/*P*	OR (95% CI)	*P* b/*P*	OR (95% CI)	*P* b/*P*	OR (95% CI)	*P* b/*P*
Total	9			2779/3484	1.09 (0.93–1.28)^c^	0.047/0.409	0.91 (0.55–1.52)^c^	<0.001/0.449	1.12 (0.92–1.37)^c^	0.002/0.271	1.03 (0.66–1.60)^c^	<0.001/0.913
Race												
Caucasian	4	0.23	0.81	693/806	1.33 (0.92–1.93)	0.053/0.130	1.72 (0.53–5.59)^c^	<0.001/0.368	1.40 (0.86–2.26)^c^	0.003/0.172	1.47 (0.53–4.08)^c^	0.001/0.458
Others	5	0.25	0.07	2086/2678	0.99 (0.87–1.12)	0.668/0.859	0.86 (0.52–1.41)^c^	0.014/0.543	0.97 (0.84–1.13)	0.270/0.708	0.86 (0.53–1.39)^c^	0.008/0.530
Country												
USA	5	0.19	0.77	1206/1745	1.18 (0.88–1.58)	0.056/0.263	1.41 (0.68–2.94)^c^	<0.001/0.357	1.28 (0.87–1.86)^c^	0.003/0.208	1.25 (0.69–2.28)^c^	0.001/0.464
Others	4	0.30	0.07	1573/1739	1.05 (0.85–1.29)	0.217/0.660	0.85 (0.38–1.90)^c^	0.008/0.699	1.03 (0.79–1.34)^c^	0.038/0.837	0.83 (0.40–1.75)^c^	0.016/0.629
Sources of controls												
Hospital-based	3	0.30	0.95	1378/1489	1.23 (0.81–1.87)^c^	0.026/0.335	1.44 (0.56–3.67)^c^	<0.001/0.453	1.33 (0.78–2.29)^c^	0.001/0.291	1.25 (0.59–2.66)^c^	<0.001/0.563
Population-based	6	0.20	0.09	1401/1995	1.06 (0.89–1.26)	0.307/0.497	0.93 (0.48–1.80)	0.256/0.041	1.05 (0.84–1.31)	0.057/0.673	0.90 (0.49–1.66)^c^	0.018/0.731
Smoke status												
Non-smokers	2	0.19	0.60	60/127	-	-	-	-	2.18 (1.13–4.19)	0.365/0.019	-	-
Smokers	2	0.17	0.95	110/220	-	-	-	-	2.66 (1.58–4.47)	0.995/<0.001	-	-

a Number of comparisons.

b *P* value of *Q*-test for heterogeneity test.

c Random-effects model was used when *P* value for heterogeneity test<0.05; otherwise, fixed-effects model was used.

**Figure 3:**
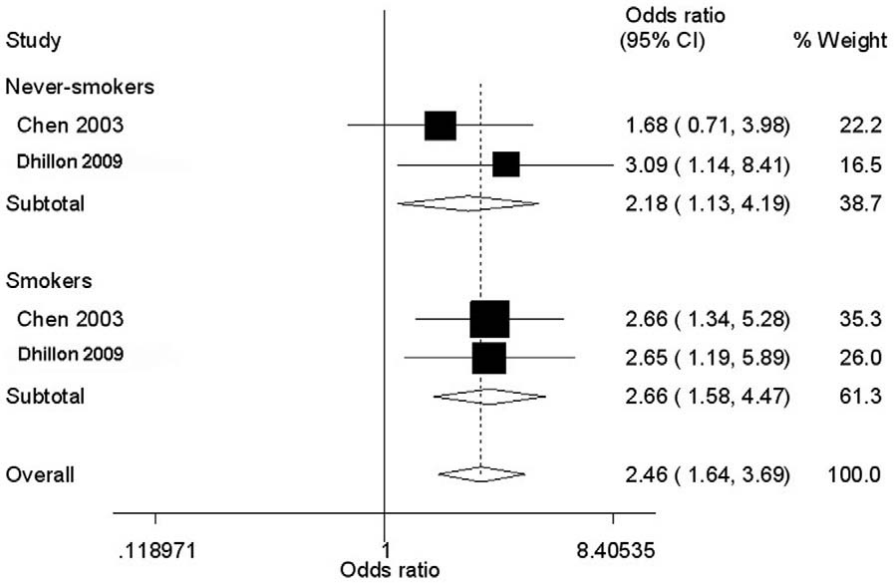
Forest plot of prostate cancer risk associated with hOGG1 polymorphism stratified by smoking status. The squares and horizontal lines correspond to the study-specific OR and 95% CI. The area of the squares reflects the weight (inverse of the variance). The diamond represents the summary OR and 95% CI.

Genotype information of the Ser^326^Cys polymorphism stratified by smoking status was available in two papers. As shown in Fig. 3, individuals with Ser/Cys+Cys/Cys genotypes showed more pronounced increased prostate cancer risk if they were smokers (OR = 2.66, 95% CI = 1.58−4.47; *P*_heterogeneity_/*P* = 0.365/0.019) rather than non-smokers (OR = 2.18, 95% CI = 1.13−4.19; *P*_heterogeneity_/*P* = 0.995/<0.001), relative to those with the Ser/Ser genotype.

### Sensitivity analysis and publication bias

**Figure 4:**
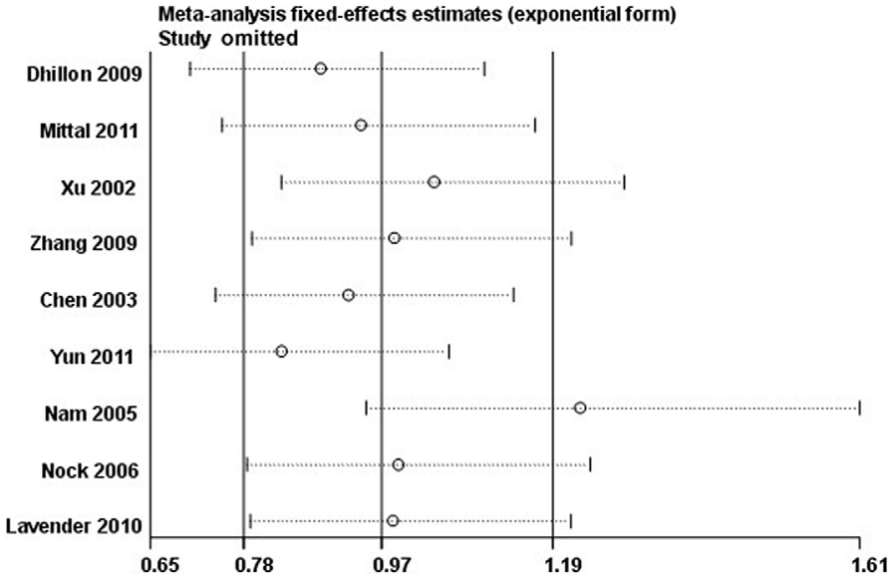
Sensitivity analysis conducted to assess the influence of each study on the pooled OR by individual studies omission.

**Figure 5:**
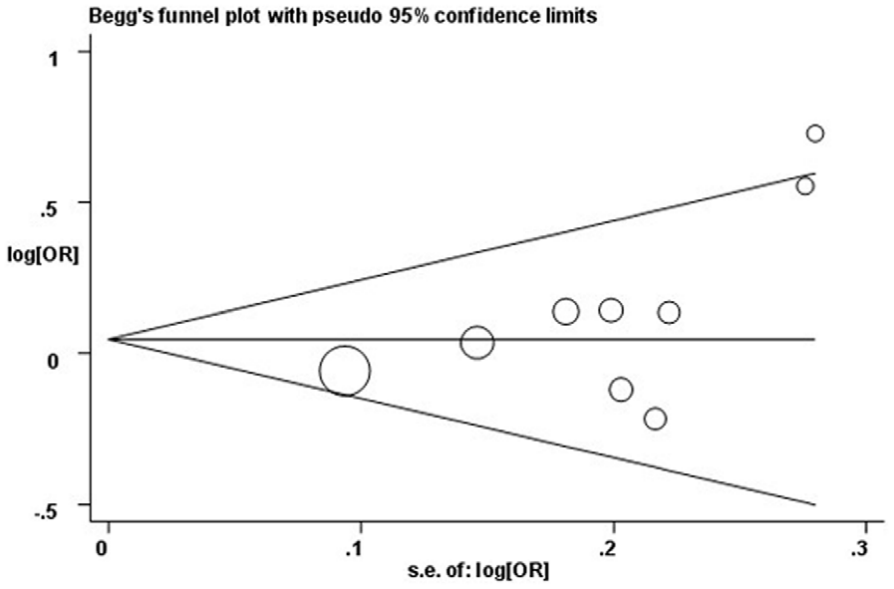
Begg's funnel plot for publication bias testing. Each point represents a separate study for the indicated association. Log[OR], natural logarithm of OR. Horizontal line, mean magnitude of the effect.

Sensitivity analysis was conducted to assess the influence of each study on the pooled OR by omission of individual studies. As shown in Fig. 4, the results suggested that no individual study would significantly affect the overall OR. Funnel plots and Egger's test (Fig. 5) were used to assess publication bias. The results indicated that there was no evidence of publication bias (*t* = 2.08, *P* = 0.076 for Ser/Cys vs. Ser/Ser).

## Discussion

The potential role of hOGG1 Ser^326^Cys polymorphism as a determinant of prostate cancer risk was investigated in a sample of 6263 subjects from nine published case-control studies. However, no significant association was found under any genetic model in the overall analysis. Ever since the hOGG1 Ser^326^Cys polymorphism was found to occur frequently in human populations, studies on this polymorphism and the risk of cancer have been conducted and published. Xu et al. first found this polymorphism to be associated with the risk of prostate cancer in a Caucasian population in 2002 [5]. After that, several investigators duplicated his work in different populations. However, the results remained confusing, even within the same population. While we were preparing the present study, a meta-analysis for this same polymorphism was published by Zhang et al. Their study included 8 papers with 2584 cases and 3234 controls, and they found an association between hOGG1 Ser^326^Cys polymorphism and prostate cancer in an African-Caucasian-mixed population. In the current study, we retrieved 9 papers with more subjects and we also stratified the analyses not only by race but also by country, source of controls, and smoking status. We found that the Cys^326^ allele was associated with a more pronounced increased risk of prostate cancer in smokers than in non-smokers.

HOGG1 is a key protein involved in base excision repair. It recognizes and excises lesions from oligodeoxynucleotides with DNA damage. Recently, studies have suggested that the hOGG1 Cys^326^ allele may be associated with an increased risk and severity of many types of cancer [13]. These results are likely to support the hypothesis that a minimally active hOGG1*Cys^326^ allozyme lacks the ability to repair the DNA damage induced by environmental chemicals in carcinogenesis. However, the exact repair function associated with this polymorphism remains unclear. Kohno et al. observed that the Cys^326^ allele was less capable of complementing a repair-deficient strain than the Ser^326^ allele, while Dherin et al. found no significant differences in hOGG1 protein enzymatic activity in vitro [Bibr pone.0030309-Kohno1] [3,14]. Janssen et al. found that DNA repair activity of hOGG1 in lymphocytes was not dependent on the Ser^326^Cys polymorphism [15]. Our results showed that the hOGG1 Cys^326^ allele was not associated with the risk of prostate cancer, confirming that the enzymatic activity of hOGG1 might not be determined by Ser^326^Cys polymorphism alone. Rather, it is possible that other confounding factors might interact with this polymorphism in the process of DNA repair.

Although cigarette smoking has been found to be involved in many cancers and is one of the leading causes of cancer-related death, its role in prostate cancer remains ill-defined [16]. Recently, a meta-analysis of 24 prospective cohort studies found smoking to be associated with higher risk of developing and dying of prostate cancer [17]. Ngo et al. also found that smoking not only independently predicted greater tumor volumes and higher grades in prostate cancer but also a greater risk of biochemical recurrence after radical prostatectomy [18]. Moreover, smoking was found to have a destructive effect on DNA [19]. In the present study, gene-environment interaction was also investigated between hOGG1 Ser^326^Cys polymorphism, cigarette smoking, and risk of prostate cancer. We found that the risk effect of Ser/Cys+Cys/Cys genotypes was more pronounced among smokers than among non-smokers. The powers of the subtype analyses were 0.60 in the non-smoker group and 0.95 in the smoker group, suggesting an interaction between the hOGG1 polymorphism and smoking with respect to prostate cancer. Such an interaction is reasonable considering that the hOGG1 protein protects cells against the reactive oxygen species and DNA adducts that can be caused by from cigarette smoking. The Cys326 allele has been reported to be associated with decreased DNA repair activity. For this reason this allele was thought to be a risk factor for smokers.

The current study has some shortcomings that should be addressed. First, the U.S., Canadian, and Australian populations were classified as Caucasian. However, populations in these areas consist both of indigenous peoples and many types of immigrants, and not all of the studies stated the ancestries of their participants clearly. Second, the controls were not uniform either. In hospital-based studies, most of the controls were BPH patients. Some individuals in the control group might be likely to develop cancer in subsequent years, even if they showed no clinical symptoms at the time of investigation. Misclassification bias can cause deviated genotype distribution in the controls. In spite of these, the present meta-analysis also had some advantages over previous studies. For the first time, we conducted a meta-analysis on hOGG1 polymorphism on prostate cancer susceptibility while considering possible confounding factors, such as smoking status, by polling the results of all published independent studies. The statistical power of the analysis is greater than that of any single study, although it remains relatively small. The quality of studies included in our meta-analysis was satisfactory and perfectly met our inclusion criteria.

In conclusion, our meta-analysis demonstrates that hOGG1 Ser^326^Cys polymorphism is a risk factor for prostate cancer in smokers. Further studies are needed to confirm the relationship. We believe that these results will foster comprehensive understanding of the association between hOGG1 polymorphism and the risk of prostate cancer with respect to gene-gene and gene-environment interactions.
